# *N*′-[(5-Methyl-2-fur­yl)methyl­ene]thio­phene-2-carbohydrazide

**DOI:** 10.1107/S1600536810010810

**Published:** 2010-03-27

**Authors:** Jin-He Jiang

**Affiliations:** a Microscale Science Institute, Department of Chemistry and Chemical Engineering, Weifang University, Weifang 261061, People’s Republic of China

## Abstract

In the title compound, C_11_H_10_N_2_O_2_S, the dihedral angle between the five-membered aromatic rings is 10.24 (12)°. In the crystal structure, mol­ecules are linked by bifurcated N—H⋯(O,N) hydrogen bonds, generating [001] chains.

## Related literature

For related structures, see: Jiang (2010*a* ▶,*b* ▶).
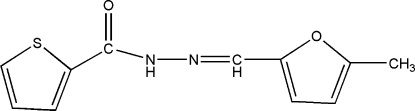


## Experimental

### 

#### Crystal data

C_11_H_10_N_2_O_2_S
*M* *_r_* = 234.27
Tetragonal, 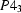

*a* = 8.8037 (12) Å
*c* = 14.670 (3) Å
*V* = 1137.0 (3) Å^3^
*Z* = 4
Mo *K*α radiation
μ = 0.27 mm^−1^
*T* = 293 K
0.25 × 0.20 × 0.19 mm

#### Data collection

Bruker SMART CCD diffractometer
10087 measured reflections
2597 independent reflections
2073 reflections with *I* > 2σ(*I*)
*R*_int_ = 0.044

#### Refinement

*R*[*F* ^2^ > 2σ(*F* ^2^)] = 0.037
*wR*(*F* ^2^) = 0.087
*S* = 0.96
2597 reflections
145 parameters
1 restraint
H-atom parameters constrained
Δρ_max_ = 0.23 e Å^−3^
Δρ_min_ = −0.14 e Å^−3^
Absolute structure: Flack (1983 ▶), 1241 Friedel pairs
Flack parameter: −0.11 (8)

### 

Data collection: *SMART* (Bruker, 1997 ▶); cell refinement: *SAINT* (Bruker, 1997 ▶); data reduction: *SAINT*; program(s) used to solve structure: *SHELXS97* (Sheldrick, 2008 ▶); program(s) used to refine structure: *SHELXL97* (Sheldrick, 2008 ▶); molecular graphics: *SHELXTL* (Sheldrick, 2008 ▶); software used to prepare material for publication: *SHELXTL*.

## Supplementary Material

Crystal structure: contains datablocks global, I. DOI: 10.1107/S1600536810010810/hb5367sup1.cif

Structure factors: contains datablocks I. DOI: 10.1107/S1600536810010810/hb5367Isup2.hkl

Additional supplementary materials:  crystallographic information; 3D view; checkCIF report

## Figures and Tables

**Table 1:** Hydrogen-bond geometry (Å, °)

*D*—H⋯*A*	*D*—H	H⋯*A*	*D*⋯*A*	*D*—H⋯*A*
N2—H2*A*⋯O1^i^	0.86	2.30	3.064 (2)	149
N2—H2*A*⋯N1^i^	0.86	2.51	3.218 (2)	140

Symmetry code: (i) 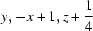.

**Fig. 1.**
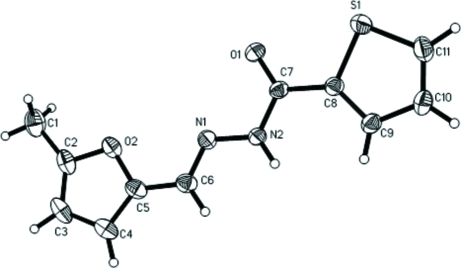
The molecular structure of (I) showing 30% probability displacement ellipsoids.

## supplementary crystallographic information

### Comment

As part of our ongoing studies of Schiff bases (Jiang, 2010a,b), we have synthesized the title compound (I), and describe its structure here, which occurs in the unusual enantiomorphous space group of P4_3_.

The molcular structure of (I) is shown in Fig. 1.   In the crystal, the molecules are linked by bifurcated N—H···(O,N) hydrogen bonds (Table 1).

### Experimental

A mixture of thiophene-2-carbohydrazide (0.05 mol) and 5-methylfuran-2-carbaldehyde (0.05 mol) was stirred in refluxing ethanol (10 ml) for 4 h to afford the title compound (0.082 mol, yield 82%). Colourless blocks of (I) were obtained by recrystallization from ethanol at room temperature.

### Refinement

H atoms were fixed geometrically and allowed to ride on their attached atoms, with C—H distances = 0.93-0.97 Å; N—H = 0.86Å and with *U*_iso_(H) = 1.2*U*_eq_(C,*N*) or 1.5*U*_eq_(C_methyl_).

### Crystal data

**Table:**

C_11_H_10_N_2_O_2_S	*D*_x_ = 1.369 Mg m^−^^3^
*M**_r_* = 234.27	Mo *K*α radiation, λ = 0.71073 Å
Tetragonal, *P*4_3_	Cell parameters from 2073 reflections
Hall symbol: P 4cw	θ = 3.3–27.5°
*a* = 8.8037 (12) Å	µ = 0.27 mm^−^^1^
*c* = 14.670 (3) Å	*T* = 293 K
*V* = 1137.0 (3) Å^3^	Block, colorless
*Z* = 4	0.25 × 0.20 × 0.19 mm
*F*(000) = 488	

### Data collection

**Table:**

Bruker SMART CCD diffractometer	2073 reflections with *I* > 2σ(*I*)
Radiation source: fine-focus sealed tube	*R*_int_ = 0.044
graphite	θ_max_ = 27.5°, θ_min_ = 3.3°
ω scans	*h* = −11→11
10087 measured reflections	*k* = −11→11
2597 independent reflections	*l* = −19→19

### Refinement

**Table:**

Refinement on *F*^2^	Secondary atom site location: difference Fourier map
Least-squares matrix: full	Hydrogen site location: inferred from neighbouring sites
*R*[*F*^2^ > 2σ(*F*^2^)] = 0.037	H-atom parameters constrained
*wR*(*F*^2^) = 0.087	*w* = 1/[σ^2^(*F*_o_^2^) + (0.0486*P*)^2^] where *P* = (*F*_o_^2^ + 2*F*_c_^2^)/3
*S* = 0.96	(Δ/σ)_max_ < 0.001
2597 reflections	Δρ_max_ = 0.23 e Å^−^^3^
145 parameters	Δρ_min_ = −0.14 e Å^−^^3^
1 restraint	Absolute structure: Flack (1983), 1241 Friedel pairs
Primary atom site location: structure-invariant direct methods	Flack parameter: −0.11 (8)

### Special details

**Table:**

Geometry. All esds (except the esd in the dihedral angle between two l.s. planes) are estimated using the full covariance matrix. The cell esds are taken into account individually in the estimation of esds in distances, angles and torsion angles; correlations between esds in cell parameters are only used when they are defined by crystal symmetry. An approximate (isotropic) treatment of cell esds is used for estimating esds involving l.s. planes.
Refinement. Refinement of *F*^2^ against ALL reflections. The weighted *R*-factor wR and goodness of fit *S* are based on *F*^2^, conventional *R*-factors *R* are based on *F*, with *F* set to zero for negative *F*^2^. The threshold expression of *F*^2^ > σ(*F*^2^) is used only for calculating *R*-factors(gt) etc. and is not relevant to the choice of reflections for refinement. *R*-factors based on *F*^2^ are statistically about twice as large as those based on *F*, and *R*- factors based on ALL data will be even larger.

### Fractional atomic coordinates and isotropic or equivalent isotropic displacement parameters (Å^2^)

**Table:**

	*x*	*y*	*z*	*U*_iso_*/*U*_eq_	
S1	0.12336 (7)	0.93062 (7)	0.06580 (4)	0.06358 (19)	
N1	0.44476 (18)	0.45913 (18)	0.09498 (10)	0.0431 (4)	
O2	0.51869 (17)	0.17921 (15)	0.03236 (10)	0.0504 (4)	
N2	0.40630 (18)	0.59910 (17)	0.12954 (10)	0.0428 (4)	
H2A	0.4438	0.6313	0.1801	0.051*	
C7	0.3074 (2)	0.6842 (2)	0.08110 (12)	0.0403 (4)	
O1	0.25704 (18)	0.64188 (17)	0.00679 (10)	0.0630 (4)	
C8	0.2645 (2)	0.8314 (2)	0.12060 (13)	0.0407 (4)	
C5	0.5748 (2)	0.2280 (2)	0.11433 (13)	0.0482 (5)	
C6	0.5305 (2)	0.3756 (2)	0.14463 (14)	0.0477 (5)	
H6A	0.5641	0.4113	0.2008	0.057*	
C9	0.3203 (2)	0.9110 (2)	0.19201 (13)	0.0477 (4)	
H9A	0.3977	0.8758	0.2296	0.057*	
C11	0.1410 (3)	1.0764 (2)	0.14106 (17)	0.0620 (6)	
H11A	0.0817	1.1638	0.1392	0.074*	
C4	0.6643 (3)	0.1185 (3)	0.15086 (18)	0.0607 (6)	
H4A	0.7162	0.1232	0.2060	0.073*	
C2	0.5746 (2)	0.0350 (2)	0.01820 (16)	0.0545 (5)	
C3	0.6633 (3)	−0.0049 (3)	0.08834 (18)	0.0658 (7)	
H3A	0.7148	−0.0965	0.0950	0.079*	
C10	0.2494 (3)	1.0520 (2)	0.20326 (15)	0.0561 (5)	
H10A	0.2747	1.1209	0.2488	0.067*	
C1	0.5204 (3)	−0.0399 (3)	−0.06593 (18)	0.0742 (7)	
H1B	0.5641	−0.1396	−0.0703	0.111*	
H1C	0.5501	0.0192	−0.1179	0.111*	
H1D	0.4117	−0.0481	−0.0642	0.111*	

### Atomic displacement parameters (Å^2^)

**Table:**

	*U*^11^	*U*^22^	*U*^33^	*U*^12^	*U*^13^	*U*^23^
S1	0.0735 (4)	0.0617 (3)	0.0555 (3)	0.0211 (3)	−0.0203 (3)	−0.0078 (3)
N1	0.0509 (9)	0.0432 (9)	0.0352 (8)	0.0052 (7)	0.0015 (6)	−0.0014 (6)
O2	0.0607 (9)	0.0452 (8)	0.0451 (8)	0.0124 (6)	0.0076 (6)	0.0052 (6)
N2	0.0528 (9)	0.0439 (8)	0.0316 (8)	0.0086 (6)	−0.0065 (7)	−0.0071 (6)
C7	0.0423 (10)	0.0458 (10)	0.0328 (10)	0.0000 (7)	−0.0028 (7)	−0.0029 (7)
O1	0.0864 (11)	0.0568 (9)	0.0458 (8)	0.0139 (8)	−0.0277 (8)	−0.0164 (7)
C8	0.0430 (10)	0.0447 (10)	0.0344 (9)	0.0018 (8)	−0.0003 (7)	−0.0019 (8)
C5	0.0488 (11)	0.0557 (12)	0.0401 (11)	0.0075 (9)	0.0064 (8)	0.0099 (9)
C6	0.0494 (11)	0.0554 (12)	0.0384 (10)	0.0056 (9)	0.0010 (8)	0.0014 (8)
C9	0.0505 (11)	0.0524 (11)	0.0402 (10)	0.0018 (9)	−0.0021 (8)	−0.0080 (8)
C11	0.0805 (16)	0.0496 (13)	0.0559 (14)	0.0165 (11)	0.0038 (12)	−0.0033 (10)
C4	0.0579 (13)	0.0622 (14)	0.0622 (14)	0.0138 (10)	−0.0010 (11)	0.0177 (11)
C2	0.0635 (13)	0.0409 (11)	0.0591 (14)	0.0078 (9)	0.0238 (11)	0.0076 (9)
C3	0.0685 (14)	0.0488 (13)	0.0800 (18)	0.0203 (11)	0.0183 (12)	0.0207 (12)
C10	0.0690 (14)	0.0488 (12)	0.0506 (13)	−0.0005 (10)	0.0065 (11)	−0.0127 (9)
C1	0.0939 (19)	0.0570 (14)	0.0716 (18)	0.0044 (13)	0.0229 (14)	−0.0038 (12)

### Geometric parameters (Å, °)

**Table:**

S1—C11	1.700 (2)	C9—C10	1.399 (3)
S1—C8	1.7187 (19)	C9—H9A	0.9300
N1—C6	1.281 (3)	C11—C10	1.338 (3)
N1—N2	1.375 (2)	C11—H11A	0.9300
O2—C5	1.369 (2)	C4—C3	1.422 (4)
O2—C2	1.377 (2)	C4—H4A	0.9300
N2—C7	1.350 (2)	C2—C3	1.339 (3)
N2—H2A	0.8600	C2—C1	1.479 (3)
C7—O1	1.234 (2)	C3—H3A	0.9300
C7—C8	1.469 (2)	C10—H10A	0.9300
C8—C9	1.353 (3)	C1—H1B	0.9600
C5—C4	1.356 (3)	C1—H1C	0.9600
C5—C6	1.428 (3)	C1—H1D	0.9600
C6—H6A	0.9300		
			
C11—S1—C8	90.80 (11)	C10—C11—S1	112.76 (17)
C6—N1—N2	116.74 (16)	C10—C11—H11A	123.6
C5—O2—C2	107.03 (16)	S1—C11—H11A	123.6
C7—N2—N1	117.51 (15)	C5—C4—C3	106.5 (2)
C7—N2—H2A	121.2	C5—C4—H4A	126.7
N1—N2—H2A	121.2	C3—C4—H4A	126.7
O1—C7—N2	121.93 (17)	C3—C2—O2	109.6 (2)
O1—C7—C8	121.47 (16)	C3—C2—C1	135.5 (2)
N2—C7—C8	116.59 (15)	O2—C2—C1	114.9 (2)
C9—C8—C7	132.01 (18)	C2—C3—C4	107.37 (19)
C9—C8—S1	111.17 (15)	C2—C3—H3A	126.3
C7—C8—S1	116.74 (13)	C4—C3—H3A	126.3
C4—C5—O2	109.5 (2)	C11—C10—C9	112.36 (19)
C4—C5—C6	133.1 (2)	C11—C10—H10A	123.8
O2—C5—C6	117.43 (17)	C9—C10—H10A	123.8
N1—C6—C5	120.43 (19)	C2—C1—H1B	109.5
N1—C6—H6A	119.8	C2—C1—H1C	109.5
C5—C6—H6A	119.8	H1B—C1—H1C	109.5
C8—C9—C10	112.89 (19)	C2—C1—H1D	109.5
C8—C9—H9A	123.6	H1B—C1—H1D	109.5
C10—C9—H9A	123.6	H1C—C1—H1D	109.5

### Hydrogen-bond geometry (Å, °)

**Table:**

*D*—H···*A*	*D*—H	H···*A*	*D*···*A*	*D*—H···*A*
N2—H2A···O1^i^	0.86	2.30	3.064 (2)	149
N2—H2A···N1^i^	0.86	2.51	3.218 (2)	140

Symmetry codes: (i) *y*, −*x*+1, *z*+1/4.
